# Effectiveness of Cardiac Rehabilitation in Exercise Capacity Increase in Patients with ST-Segment Elevation Myocardial Infarction

**DOI:** 10.3390/ijerph16214085

**Published:** 2019-10-24

**Authors:** Anna Kasperowicz, Maciej Cymerys, Tomasz Kasperowicz

**Affiliations:** 1 Cardiovascular Research Centre, University of Zielona Gora, 65-046 Zielona Gora, Poland; 2 Department of Internal Medicine, Poznan University of Medical Sciences, 61-701 Poznań, Poland; maciejcymerys@wp.pl; 3 ADB Polska Sp. z o.o., Zielona Góra office, 65-119 Zielona Góra, Poland; kasper13@interia.pl

**Keywords:** STEMI, cardiac rehabilitation, exercise capacity

## Abstract

*Background*: The efficacy of interventions in ST-segment elevation myocardial infarction (STEMI) assessed by a decrease in inpatient mortality in Poland is very high. However, a rise in mortality rate is recorded within 3 years of the discharge from the intervention centre. In order to reduce out-of-hospital mortality, the treatment should be continued with cardiac rehabilitation after hospitalization. The aim of this retrospective study was to evaluate the effect of cardiac rehabilitation on exercise capacity increase patients with STEMI with regard to their age, gender, Body Mass Index (BMI), ejection fraction (EF), concomitant diabetes and nicotine dependence. The effectiveness of cardiac rehabilitation was assessed by exercise ECG (electrocardiogram) stress test or the 6-min walk test, prior to and after cardiac rehabilitation completion. *Methods*: The study group included 100 randomly selected patients undergoing cardiac rehabilitation after STEMI, aged 40–75 years, with BMI ≤ 40 kg/m^2^, with controlled arterial hypertension, without anemia and any pulmonary comorbidities. *Results*: The study patients’ exercise capacity was observed to have increased significantly (+1 metabolic equivalent (MET) in exercise ECG stress test and +75.4 m in the 6-min walk test) regardless of their gender, age, BMI and nicotine dependence. *Conclusions*: This study proved that every patient with STEMI could benefit from cardiac rehabilitation. Nicotine-dependents, males, patients aged ≤55 and those with reduced EF (<50%) were found to have benefitted most substantially.

## 1. Introduction

Currently, coronary heart disease is the most common cause of death in the world. Annually, over 8.76 million people die of coronary disease, which constitutes 15.5% of all deaths [1]. In-hospital mortality falls within the range of 3.5–14% [2]. Around 12% of patients die within 6 months of hospital discharge [3]. Recent years have seen a decrease in in-hospital mortality caused by STEMI, however, late mortality in post-infarct patients which increases significantly within 3 years after hospital discharge still poses a serious problem [4,5].

A patient with extensive myocardial infarction who leaves hospital should undergo comprehensive medical care, which is composed of outpatient care, post-hospital rehabilitation, optimisation of pharmacotherapy, ‘planned’ revascularization, electrotherapy and secondary prevention.

A reduction in late post-infarct mortality is an undeniable advantage of rehabilitation [6,7].

The guidelines of European Society of Cardiology (ESC) of 2012 on the treatment of patients with STEMI refer to the meta-analysis, which showed that exercise training as the basic element of cardiac rehabilitation programmes was connected with a reduced risk of cardiovascular deaths in coronary heart disease patients by 26% [6,8]. An increase of exercise capacity by 1 metabolic equivalent (MET) has also been proven to reduce all-cause mortality by 8% to 14% [7].

According to ESC Guidelines of 2016, patients after Myocardial Infarction (MI), Coronary Artery Bypass Surgery (CABG) or Percutaneous Coronary Interventions (PCI), with stable angina pectoris or stable Heart Failure (HF) should perform aerobic exercise training of moderate up to high intensity for a minimum of 30 min at least 3 times a week [9,10]. It has been estimated that an increase in exercise capacity by 1 MET results in a fall in mortality rate by mean 26% [6]. Furthermore, the distance increase by >70 m in the 6-min walk test may be indicative of significantly improved exercise capacity [11].

Regular aerobic exercise leads to a diminished heart muscle demand for oxygen, which reduces the risk of myocardial ischemia [12]. Exercise enhances myocardial perfusion via vasodilation (vascular relaxation), microvascular system development and improved endothelial functioning [13,14]. Other advantages of regular physical activity include a decrease in pro-thrombotic activity, blood viscosity and platelet aggregation as well as an increase in thrombolytic activity [15].

The aim of this retrospective study was to evaluate the effect of cardiac rehabilitation on exercise capacity (estimated by exercise ECG stress test or the 6-min walk test) in patients with ST-elevated myocardial infarction (STEMI) with regard to their gender, age, Body Mass Index (BMI), ejection fraction (EF) and concomitant diabetes and nicotine dependence.

The study assessment was designed to verify whether the examined population had significantly improved their exercise capacity and to determine the group of patients who benefitted most significantly from cardiac rehabilitation which was indicated by the exercise capacity increase. There was no control group in this study as our study design should require the controls to include patients after cardiovascular incidents who did not participate in a post-infarct rehabilitation programme. Creating such a group of patients could be considered as unethical and potentially qualified as medical malpractice. Additionally, as cardiac rehabilitation is not performed in healthy people there are not available data from studies or programmes which have compared the effectiveness or the effects of cardiac rehabilitation on exercise capacity in healthy people vs. cardiovascular patients.

## 2. Materials and Methods

The study protocol was approved by the Bioethical Commission at Karol Marcinkowski Medical University in Poznan (Decision No 947/15 of 5 November 2015). A retrospective chart review was undertaken to identify 100 patients at Lubuskie Pulmonology and Cardiology Hospital in Torzym who were hospitalized at Cardiac Rehabilitation Department in 2005–2015, and met the following criteria:

- age 40–75 years, after ST-segment elevation myocardial infarction treated with invasive procedures (after complete revascularization) who participated in post-infarct rehabilitation programme,
- BMI ≤40 kg/m^2^,
- ECG record with determined EF value,
- controlled arterial hypertension (baseline blood pressure in exercise test ≤ 140/90 mmHg),
- no anemia (Hb ≥ 11 g%),
- no pulmonary comorbidities (asthma, acute chronic obstructive pulmonary disease (COPD)),
- a minimum 3-week hospitalization at the cardiac rehabilitation department with exercise ECG test or the 6-min walk test performed at least twice (prior to and after rehabilitation).

The patients’ exercise capacity was evaluated by means of exercise ECG test or the 6-min walk test. The test selection was based on the general assessment of risk and baseline capacity. The patients with exercise capacity estimated as low (<4 MET) in medical history were assessed in the 6-min walk test while the others underwent the exercise ECG test on a treadmill according to Bruce Protocol.

In order to identify the patients who benefitted most considerably from cardiac rehabilitation they were allocated to subgroups according to gender, age (≤55, >55 (years)), BMI (<25, ≥25 (kg/m^2^)), EF (<40, 40–49, ≥50 (%)), concomitant diabetes or nicotine dependence.

Statistical analysis was made in R language, version 3.3.3, Windows 8.1 [16]. Wilcoxon signed-rank test was used to determine whether the increase in exercise capacity was statistically significant. In order to determine whether the subgroups differed within the analysed characteristics Mann–Whitney U test was applied. *p* value < 0.05 was considered as statistically significant.

Characteristics of the study population is shown in Table 1.

## 3. Results

In the analysed population a statistically significant increase in exercise capacity was observed in both tests. The rise in exercise capacity by +1.0 MET (from 7.2 ± 2.0 to 8.2 ± 1.9, *p* < 0.001) was identified in the patients qualified for exercise ECG test (*n* = 59) whereas in the subjects who performed the 6-min walk test (*n* = 41) an increase in exercise capacity amounted to +75.4 m (from 380.2 ± 95.9 to 455.6 ± 103.5, *p* < 0.001).

In the study of the following subgroups: nicotine-dependents, males, patients aged ≤55 years and people with reduced EF (<50%) were observed to have gained the most considerable benefits from cardiac rehabilitation, which was reflected in an exercise capacity increase.

The test results and the subgroup characteristics are presented in Table 2 and Table 3 respectively.

In our study population no statistically significant differences in the subgroups were found (Table 4).

### 3.1. Gender

The study included 40 female and 60 male patients. In exercise ECG stress test a statistically significant improvement in exercise capacity was observed in both groups: +0.7 MET in females (*n* = 27) and +1.2 MET in males (*n* = 32). In the 6-min walk test both groups were found to have an elevated exercise capacity level: +56.8 m in females (*n* = 13) and +84.1 m in males. A more substantial absolute increase in exercise capacity was found in males despite no statistically significant differences between the analysed groups.

### 3.2. Age

In the study age groups, a statistically significant increase in exercise capacity was identified in all of the assessed subgroups regardless of the test they performed. In the exercise ECG stress test, the exercise capacity was found to have risen by +1.2 MET in age group ≤55 years (*n* = 27) and +0.8 MET in age group >55 years (*n* = 32). In the 6-min walk test, the age group ≤55 years (*n* = 7) showed an increase in their exercise capacity by +117.3 m in comparison with the age group >55 years (*n* = 34) with ane increase of +66.8 m. A more substantial absolute increase in exercise capacity was found in the age group ≤55 years although no statistically significant differences were found between the analysed groups.

### 3.3. BMI

Owing to the accepted BMI criteria, no statistically significant increase in exercise capacity was observed in exercise ECG test in the group of BMI < 25 kg/m^2^ (*n* = 14) (+0.4 MET, *p* = 0.126) although in the group of BMI ≥ 25 kg/m^2^ (*n* = 45) in the same test the increase in exercise capacity (+1.2 MET) was statistically significant. In the study patients qualified for the 6-min walk test an increase in exercise capacity was found in both subgroups: +86.0 m in BMI < 25 kg/m^2^ (*n* = 6) and +73.6 m in BMI ≥ 25 kg/m^2^ (*n* = 35). The obtained results are not sufficiently compelling to conclude which group benefitted most substantially from cardiac rehabilitation. No statistically significant differences were observed between the analysed subgroups.

### 3.4. EF

The patients were divided into three groups with regard to EF values: EF < 40% (*n* = 26), EF 40–49% (*n* = 31) and EF ≥ 50% (*n* = 43). In the exercise ECG stress test, a statistically significant increase in exercise capacity +1.4 MET and +0.7 MET was recorded in the following two groups: EF 40–49% (*n* = 23) and EF ≥ 50% (*n* = 36) respectively. In our study population no patients with EF < 40% were qualified for exercise ECG stress test. In the 6-min walk test the study patients with EF < 40% (*n* = 26) and EF 40–49% (*n* = 8) were found to have increased their exercise capacity significantly by +80.6 m and +66.6 m respectively. No statistically significant rise in exercise capacity was recorded in the group with EF ≥50% (*n* = 7) (+65.7 m, *p* = 0.297). A more considerable increase in absolute values of exercise capacity was found in the group with EF < 50% despite no statistically significant differences between the analysed groups.

### 3.5. Diabetes

In the exercise ECG stress test, exercise capacity in diabetic patients (*n* = 19) was found to have increased statistically insignificantly (+0.5 MET, *p* = 0.065). The same test performed by the patients without concomitant diabetes (*n* = 40) revealed an increase in exercise capacity by +1.2 MET which was statistically significant. In the 6-min walk test, a statistically significant increase in exercise capacity was observed in both subgroups: +86.8 m in diabetic patients (*n* = 21) and +63.5 m in non-diabetics (*n* = 20). The obtained results are not unequivocal enough to determine which subgroup gained more benefits from rehabilitation. No statistically significant differences were found between the analysed subgroups.

### 3.6. Nicotine Dependence

When the patients were divided into groups according to their nicotine dependence, a statistically significant improvement in exercise capacity was recorded in all analysed subgroups, regardless of the test they underwent. In exercise ECG stress test the exercise capacity increased by +1.2 MET in nicotine-dependent group (*n* = 30) in comparison to the increase of +0.74 MET in non-smokers (*n* = 29). In the 6-min walk test, the exercise capacity was observed to have increased by +77.5 m in smokers (*n* = 18) compared to the rise by 73.8 m in non-smokers (*n* = 23). A more considerable increase in absolute values of exercise capacity was found in nicotine-dependent group although no statistically significant differences between the analysed groups were noted.

## 4. Discussion

Cardiovascular diseases are the most common cause of death worldwide [17]. Although enormous progress has recently been made in invasive procedures of acute coronary syndrome treatment, post-hospital mortality is still high, which may partly be ascribed to the lack of comprehensive cardiac rehabilitation or its insufficient availability [4,5]. The exercise ECG stress test and the 6-min walk tests are applied as the criteria to assess the effectiveness of cardiac rehabilitation in the exercise capacity improvement.

A study conducted in a group of 57 post-infarct males aged 54.5 ± 7.0 years and post-infarct 30 females aged 52.0 ± 6.7 years, who underwent 8-week rehabilitation, reported a significant increase in exercise capacity regardless of gender but the rate of males who returned to work was higher (78.9%) when compared to females (50.0%) [18]. In another similar study, both males (*n* = 41) and females (*n* = 42) were observed to have improved their exercise capacity statistically significantly after post-infarct rehabilitation [19]. The abovementioned outcomes are consistent with the results of this study which proved that gender was not the factor which affected the effectiveness of rehabilitation

The impact of cardiac rehabilitation with regard to age (40–80 years) and gender was analysed in a group of males (*n* = 41) and females (*n* = 42). In the study patients, the males over 70 years old were the only ones whose exercise capacity improvement did not reach statistical significance [19]. In our study the age criterium ≤55 and >55 years of age was used without gender differentiation. In both age groups a statistically significant increase in exercise capacity was observed.

The relation of BMI and cardiac rehabilitation outcomes was analysed in two groups of patients: BMI ≥ 25 kg/m^2^ (*n* = 170) and BMI < 25 kg/m^2^ (*n* = 189). Both groups proved to have improved their capacity in the exercise test without a statistically significant difference between groups [20]. This study showed that patients with BMI ≥ 25 kg/m^2^ proved their exercise capacity increase in both exercise tests whereas patients with BMI < 25 kg/m^2^ achieved a statistically significant increase only in the 6-min walk test.

A relation between left ventricular dysfunction and an increased survival rate connected with exercise training was recorded in the meta-analysis of 2004 [21]. Better prognosis was also reported in a study of 2009 where the patients compliance with the recommended exercise intensity was the crucial element of the exercise capacity improvement [22]. Twelve percent mortality rate reduction linked to the exercise capacity increase by 1 MET was shown in a group of males (*n* = 6213) who were divided into patients with abnormal exercise test results and/or with diagnosed cardiovascular disease (*n* = 3679) and patients with normal exercise test results and without cardiovascular diseases (*n* = 2534) [7]. The increase in exercise capacity appeared to be the most substantial in the patients (*n* = 129) with the lowest exercise capacity (measured by oxygen intake pVO_2_ < 20 mL/kg/min) [23]. Appropriate physical training performed by patients with heart failure does not only increase the exercise capacity (measured by peak oxygen intake) but also improves the quality of life, thereby potentially decreasing the frequency of rehospitalization [24]. This study results are consistent with the outcomes of the abovementioned papers: the analysed patients were shown to have significantly increased their exercise capacity. The most considerable increase in the capacity was observed in patients with severe left ventricular systolic dysfunction (EF < 40%) in the 6-min walk test. It should be underlined that none of the analysed patients with EF < 40% were qualified for the exercise ECG stress test while in the 6-min walk test the study patients with EF ≥ 50% (*n* = 7) did not show a statistically significant increase in their exercise capacity.

Exercise capacity improvement after 12-week cardiac rehabilitation was investigated in diabetics (*n* = 370) and non-diabetic patients (*n* = 942) [25]. A statistically significant increase in exercise capacity was observed in both groups, with the increase being less considerable in the diabetics. In yet another study both male and female diabetics were investigated (7036 non-diabetic and 1546 diabetic patients underwent cardiac rehabilitation, 12-week rehabilitation was completed by 7173 patients (5973 non-diabetics and 1230 diabetics)) [26]. After cardiac rehabilitation, an increase in exercise capacity was observed in all groups: +1.0 MET in non-diabetic males, +0.9 MET in diabetic males and non-diabetic females, +0.7 MET in diabetic females, without statistically significant differences between the analysed groups. The abovementioned outcomes are consistent with the results of this study (except ambiguous results for diabetic patients in the 6-Minute Walk Test). A small-scale study (*n* = 37) was also conducted to compare the improvement in exercise capacity after post-MI cardiovascular rehabilitation in diabetic (*n* = 12) and non-diabetic (*n* = 25) patients (aged over 30 and below 70 years) who had participated in ECG-monitored exercise at least four to eight times in eight weeks of cardiac rehabilitation [27]. The study results indicated significantly improved METs (one of the measured factors) after cardiac rehabilitation in both groups with diabetics showing significantly lower METs than the non-diabetic group. In our study the outcomes for patients with and without concomitant diabetes were found to be ambiguous. In the ECG Stress Test a statistically significant improvement in exercise capacity was proven in patients without concomitant diabetes (+1.2 MET, *p* < 0.001, *n* = 40) but no statistically significant increase was observed in diabetic patients (+0.5, *p* = 0.065, *n* = 19). In the 6-Minute Walk Test a statistically significant improvement was recorded in both groups of patients but in patients with concomitant diabetes the increase was more considerable (+86.8 m, *p* < 0.001, *n* = 21) compared with the non-diabetic group (+63.4, *p* < 0.001, *n* = 20). Due to the abovementioned discrepancies in achieved outcomes, an unambiguous assessment of the effectiveness of the cardiac rehabilitation may be difficult to evaluate with regard to diabetes. Further studies are needed to assess if there are more concomitant factors which might have an impact while defining the exact relation between cardiac rehabilitation and diabetes.

The available literature does not provide unequivocal results of the studies on the impact of concomitant nicotine-dependence on the exercise capacity improvement in cardiac rehabilitation patients. Tobacco smoking produces a strong pro-thrombotic effect. The risk of STEMI is twice as high in smokers [28,29]. Tobacco smoking cessation is the most effective method of secondary prevention [30]. According to this study outcomes, both nicotine-dependent patients and non-smokers improved their exercise capacity which was particularly detectable in exercise ECG test. Additionally, in our study, patients from nicotine-dependent group have improved their exercise capacity in both types of tests, but without a statistically significant difference when compared with non-smokers. This might be the result of smoking cessation and the simultaneous start of physical activity. Nevertheless, further studies are needed to investigate a potential relation between nicotinism and cardiac rehabilitation.

In this study there was no control group. The study population was recruited from one centre. Patients with non-ST-elevation myocardial infarction were excluded from the study as many clinical situations can produce the symptoms of acute coronary syndrome without changes in coronary arteries. The study included the patients with controlled arterial hypertension (up to 140/90 mmHg) in order to exclude potential events when high blood pressure symptoms prevail during exercise test. Our study focused mostly on the results of three weeks’ cardiac rehabilitation. No data was available on the results after a long-term follow-up as this could be obtained only in patients who have regularly been re-hospitalized.

## 5. Conclusions

Patients with ST-segment elevation myocardial infarction who undergo cardiac rehabilitation programme are observed to increase their exercise capacity significantly. The increase occurs regardless of gender, age, BMI and nicotine-dependence. Exercise ECG stress test and the 6-min walk test alike are applied to assess the exercise capacity improvement. A three-week in-hospital cardiac rehabilitation produced the most beneficial effects in nicotine-dependents, males, patients aged ≤55 years and those with reduced EF (<50%).

## Figures and Tables

**Table 1:** Characteristics of examined population.

Parameters		*n*	Gender		Age (years)	BMI (kg/m^2^)	EF (%)	D+ (*n*)	N+ (*n*)	ECGST (*n*)	6MWT (*n*)
			Women	Men							
Population		100	40	60	58.3 ± 7.8	28.6 ± 4.9	45.9 ±11.0	40	48	59	41
Gender	Women	40	40	0	58.6 ± 7.2	28.3 ± 5.7	50.4 ±7.2	16	19	27	13
	Men	60	0	60	58.1 ± 8.2	28.8 ± 4.3	42.9 ± 12.1	24	29	32	28
Age (years)	≤55	34	13	21	49.4 ± 4.3	28.8 ± 5.0	47.4 ± 9.1	14	22	27	7
	>55	66	27	39	62.9 ± 4.6	28.5 ± 4.9	45.2 ± 11.9	26	26	32	34
BMI (kg/m^2^)	<25	20	11	9	57.8 ± 7.1	22.4 ± 1.7	45.9 ± 11.0	2	16	14	6
	≥25	80	29	51	58.4 ± 8.0	30.2 ± 4.1	45.9 ± 11.1	38	32	45	35
EF (%)	<40	26	3	23	61.5 ± 7.2	28.9 ± 4.8	31.0 ± 5.4	13	13	0	26
	40–49	31	14	17	56.8 ± 8.4	28.1 ± 5.5	44.7 ± 2.9	11	16	23	8
	≥50	43	23	20	57.4 ± 7.2	28.8 ± 4.5	55.8 ± 5.0	16	19	36	7
Diabetes (*n*)	D+	40	16	24	59.3 ± 7.7	30.0 ± 4.5	44.8 ± 10.1	40	16	19	21
	D−	60	24	36	57.6 ± 7.8	27.7 ± 4.9	46.6 ± 11.7	0	32	40	20
Nicotinism (*n*)	N+	48	19	29	56.2 ± 7.6	27.8 ± 5.3	45.4 ± 10.8	16	48	30	18
	N−	52	21	31	60.2 ± 7.5	29.4 ± 4.4	46.4 ± 11.3	24	0	29	23

D+: with concomitant diabetes, D−: without concomitant diabetes, N+: with concomitant nicotinism, N−: without concomitant nicotinism, ECGST: ElectroCardioGram Stress Test, 6MWT: the 6-Minute Walk Test.

**Table 2:** ECG Stress Test population and results.

ECGST		*n*	Gender		Age (years)	BMI (kg/m^2^)	EF (%)	D+ (*n*)	N+ (*n*)	Before (MET)	After (MET)	Diff (MET)	*p*-Value
			Women	Men									
Population		59	27	32	55.9 ± 7.8	28.1 ± 4.6	51.7 ± 7.2	19	30	7.2 ± 2.0	8.2 ± 1.9	1.0	<0.001
Gender	Women	27	27	0	56.5 ±7.0	27.3 ± 5.2	51.9 ± 6.7	8	15	6.3 ± 1.7	7.0 ± 1.7	0.7	0.003
	Men	32	0	32	55.4 ± 8.5	28.9 ± 4.0	51.6 ± 7.7	11	15	8.0 ± 1.8	9.2 ± 1.4	1.2	<0.001
Age (years)	≤55	27	11	16	48.7 ± 4.4	28.4 ± 4.7	49.9 ± 7.0	7	18	7.2 ± 2.2	8.4 ± 2.0	1.2	<0.001
	>55	32	16	16	61.9 ± 3.8	27.9 ± 4.5	53.3 ± 7.1	12	12	7.2 ± 1.7	8.0 ± 1.8	0.8	0.002
BMI (kg/m^2^)	<25	14	10	4	56.0 ± 7.2	22.4 ± 1.7	50.8 ± 7.9	2	11	7.5 ± 1.7	7.9 ± 1.7	0.4	0.126
	≥25	45	17	28	55.9 ± 8.0	29.9 ± 3.7	52.0 ± 7.0	17	19	7.1 ± 2.1	8.3 ± 2.0	1.2	<0.001
EF (%)	<40	0	-	-	-	-	-	-	-	-	-	-	-
	40–49	23	10	13	54.4 ± 8.0	27.5 ± 4.9	44.5 ± 3.0	7	13	6.8 ± 2.0	8.2 ± 1.8	1.4	<0.001
	≥50	36	17	19	56.9 ± 7.6	28.6 ± 4.5	56.3 ± 5.0	12	17	7.5 ± 1.9	8.2 ± 2.0	0.7	0.002
Diabetes (*n*)	D+	19	8	11	56.9 ± 7.1	29.2 ± 4.7	51.5 ± 6.2	19	8	7.2 ± 2.0	7.7 ± 2.2	0.5	0.065
	D−	40	19	21	55.4 ± 8.1	27.6 ± 4.5	51.8 ± 7.7	0	22	7.2 ± 2.0	8.4 ± 1.7	1.2	<0.001
Nicotinism (*n*)	N+	30	15	15	54.2 ± 7.8	27.4 ± 4.6	51.0 ± 7.4	8	30	7.0 ± 1.9	8.2 ± 1.7	1.2	<0.001
	N−	29	12	17	57.6 ± 7.4	28.9 ± 4.6	52.4 ± 7.0	11	0	7.5 ± 2.0	8.2 ± 2.1	0.7	0.008

D+: with concomitant diabetes, D−: without concomitant diabetes, N+: with concomitant nicotinism, N−: without concomitant nicotinism, ECGST: ElectroCardioGram Stress Test, before: average (mean and sd) value of ECGST test before rehabilitation, after: average (mean and sd) value of ECGST after rehabilitation, diff: difference before/after rehabilitation, *p*-value: *p*-value of Wilcoxon Test to measure if difference (diff) is statistically significant (*p* < 0.05).

**Table 3:** 6-Minute Walk Test population and results.

6MWT		*n*	Gender		Age (years)	BMI (kg/m^2^)	EF (%)	D+ (*n*)	N+ (*n*)	Before (m)	After (m)	Diff (m)	*p*-Value
			Women	Men									
Population		41	13	28	61.8 ± 6.5	29.3 ± 5.2	37.5 ± 10.2	21	18	380.2 ± 95.9	455.6 ± 103.5	75.4	<0.001
Gender	Women	13	13	0	62.9 ± 5.9	30.5 ± 6.2	47.4 ± 7.6	8	4	330.3 ± 91.6	387.1 ± 74.8	56.8	0.023
	Men	28	0	28	61.2 ± 6.8	28.8 ± 4.7	33.0 ± 7.8	13	14	403.3 ± 90.2	487.4 ± 100.4	84.1	<0.001
Age (years)	≤55	7	2	5	52.1 ± 2.9	30.3 ± 6.0	37.7 ± 10.5	7	4	352.1 ± 132.0	469.4 ± 141.7	117.3	0.016
	>55	34	11	23	63.7 ± 5.1	29.1 ± 5.1	37.5 ± 10.3	14	14	385.9 ± 88.1	452.7 ± 96.4	66.8	<0.001
BMI (kg/m^2^)	<25	6	1	5	61.8 ± 5.2	22.2 ± 1.8	34.5 ± 8.6	0	5	457.7 ± 41.8	543.7 ± 25.8	86.0	0.031
	≥25	35	12	23	61.7 ± 6.7	30.5 ± 4.6	38.1 ± 10.5	21	13	366.9 ± 96.5	440.5 ± 104.4	73.6	<0.001
EF (%)	<40	26	3	23	61.5 ± 7.2	28.9 ± 4.8	31.0 ± 5.4	13	13	384.7 ± 94.2	465.3 ± 104.2	80.6	<0.001
	40–49	8	4	4	63.8 ± 5.4	29.9 ± 7.1	45.4 ± 2.4	4	3	396.5 ± 68.0	463.1 ± 84.6	66.6	0.022
	≥50	7	6	1	60.3 ± 4.7	30.1 ± 4.9	53.0 ± 4.4	4	2	344.9 ± 130.5	410.6 ± 122.3	65.7	0.297
Diabetes (n)	D+	21	8	13	61.5 ± 7.7	30.7 ± 4.4	38.8 ± 9.0	21	8	350.3 ± 95.7	437.1 ± 99.3	86.8	<0.001
	D−	20	5	15	62.0 ± 5.1	27.9 ± 5.7	36.2 ± 11.4	0	10	411.5 ± 87.7	474.9 ± 106.8	63.4	<0.001
Nicotinism (*n*)	N+	18	4	14	59.4 ± 6.1	28.6 ± 6.4	35.9 ± 8.8	8	18	396.9 ± 73.3	474.4 ± 103.7	77.5	<0.001
	N−	23	9	14	63.6 ± 6.3	29.9 ± 4.1	38.8 ± 11.2	13	0	367.0 ± 110.2	440.8 ± 103.2	73.8	<0.001

D+: with concomitant diabetes, D−: without concomitant diabetes, N+: with concomitant nicotinism, N−: without concomitant nicotinism, 6MWT: the 6-Minute Walk Test, before: average (mean and sd) value of 6MWT test before rehabilitation, after: average (mean and sd) value of 6MWT after rehabilitation, diff: difference before/after rehabilitation, *p*-value: *p*-value of Wilcoxon Test to measure if difference (diff) is statistically significant (*p* < 0.05).

**Table 4:** Differences between groups (Mann–Whitney U Test).

Examined Groups	ECGST	6MWT
Gender (Women, Men)	no differences, *p* = 0.293	no differences, *p* = 0.223
Age (years) (≤55, >55)	no differences, *p* = 0.166	no differences, *p* = 0.232
BMI (kg/m^2^) (<25, ≥25)	no differences, *p* = 0.170	no differences, *p* = 0.406
EF (%) (<40, 40–49)	not enough data	no differences, *p* = 0.776
EF (%) (<40, ≥50)	not enough data	no differences, *p* = 0.567
EF (%) (40–49, ≥50)	no differences, *p* = 0.101	no differences, *p* = 0.772
Diabetes (D+, D−)	no differences, *p* = 0.110	no differences, *p* = 0.404
Nicotinism (N+, N−)	no differences, *p* = 0.172	no differences, *p* = 0.916

D+: with concomitant diabetes, D−: without concomitant diabetes, N+: with concomitant nicotinism, N−: without concomitant nicotinism, ECGST: ElectroCardioGram Stress Test, 6MWT: the 6-Minute Walk Test, *p*: *p*-value of Mann–Whitney U Test to measure if the difference between groups is statistically significant (*p* < 0.05).
